# COVID-19 and BRD4: a stormy and cardiotoxic bromo-romance

**DOI:** 10.20517/jca.2021.20

**Published:** 2022-01-01

**Authors:** Emma L. Robinson, Timothy A. McKinsey

**Affiliations:** Department of Medicine, Division of Cardiology and Consortium for Fibrosis Research & Translation, University of Colorado Anschutz Medical Campus, Aurora, CO 80045-0508, USA.

**Keywords:** SARS-CoV-2, COVID-19, cytokine storm, bromodomain-containing protein 4, diastolic dysfunction

## Abstract

Severe systemic inflammation in COVID-19 patients can lead to dysfunction of multiple organs, including the heart. Using an ex vivo cardiac organoid system, Mills et al discovered that inhibitors of the chromatin reader protein, bromodomain-containing protein 4, protect cardiomyocytes from COVID-associated “cytokine storm”. We briefly review these important findings and highlight the translational significance of the work.

COVID-19 disease, caused by the severe acute respiratory syndrome (SARS)-like coronavirus (SARS-CoV-2), affects multiple organs with an unusual degree of heterogeneity in the form and severity of acute symptoms between infected individuals. To date, SARS-CoV-2 infection is estimated to have been responsible for nearly 4.5 million deaths, with at least 213 million individuals infected, 100-fold more than the annual mortality rate from the common influenza virus. Moreover, given the sudden recent onset of this destructive and highly contagious infectious agent, long-term implications on human health post-infection are still being unveiled.

Cardiac involvement in COVID-19 was recognized early in the pandemic, with elevated circulating markers of heart damage found in many patients. COVID-19 patients most commonly present with right ventricular dilation and dysfunction, although defects in left ventricular (LV) performance are also observed; LV diastolic dysfunction (DD) outweighs systolic dysfunction in these individuals^[1]^. Cardiomyocytes express the receptor for SARS-CoV-2, angiotensin-converting enzyme 2 (ACE2), which is upregulated in heart failure^[2]^. While the ability of SARS-CoV-2 to directly infect cardiomyocytes has been demonstrated by evaluation of a small set of autopsy and endomyocardial biopsy specimens^[3,4]^, indirect, secondary effects of infection likely serve a more significant role in eliciting cardiac complications. In particular, the pro-inflammatory state-the so-called cytokine storm (CS)-associated with COVID-19 is thought to be a major cause of multi-organ morbidity and failure and poor prognosis of severe infection.

In a tour de force study recently published in *Cell*, Mills *et al.*^[5]^ tackled the problem of CS-induced cardiac damage. The authors initially employed human pluripotent stem cell-derived cardiac organoids (hCOs) in culture to screen different combinations of cytokines and other factors for effects on contractile force or relaxation. Tumor necrosis factor (TNF) treatment led to impaired systolic function of hCOs, whereas the combination of IFN-γ, IL-1β, and dsDNA [poly(I:C)] induced the most severe DD phenotype, causing a ~50% increase in hCO relaxation time. This DD-inducing mixture was selected to advance into further *in vitro* studies to mimic CS in COVID-19 patients.

To address the mechanism of CS-mediated cardiac damage, high sensitivity phosphoproteomics analysis was performed, which revealed a novel and major shift with CS in hCOs, with induction of phosphorylation of many proteins that are critical for the regulation of cardiac function, including signaling effectors (e.g., GRK2 and PKA), as well as transcriptional regulators [e.g., MEF2A, STAT1 and bromodomain-containing protein 4 (BRD4)]. A chemical biology approach with inhibitors of top candidates from the phospho-screen was employed to test for compounds that can counteract TNF and CS-induced systolic dysfunction and DD, respectively. The most promising relaxation-inducing “hit” from this mini-screen was INCB054329, which inhibits BRD4, as well as the three other members of the bromodomain and extra-terminal (BET) acetyl-lysine reader protein family, BRD2, BRD3, and the testis-specific BRDT, all of which have two tandem N-terminal acetyl-lysine recognition motifs, or bromodomains (BD1 and BD2). INCB054329 also improved the relaxation of hCOs treated with serum from a COVID-19 patient as the source of CS. INCB054329 binds to BD1 and BD2 of BET proteins, thereby functioning as a competitive inhibitor that displaces the readers from acetyl-histones on chromatin. Prior studies showed that a related BET inhibitor, JQ1, improves systolic function, with associated reductions in cardiac hypertrophy, fibrosis, and inflammation, in mouse models of heart failure^[6]^. However, the Mills paper is the first to reveal the potential utility of BET inhibitors for the treatment of DD.

Since mice cannot be infected by SARS-CoV-2, follow-up *in vivo* studies were performed with “humanized” mice harboring a transgene for human ACE2^[7]^. Bulk RNA-seq of hearts of mice infected with SARS-CoV-2 (96 h total) and treated with INCB054329 or vehicle control at 24, 48, and 72 h post-infection, showed that the BET inhibitor suppressed expression of > 20% of genes upregulated by the virus. Furthermore, SARSCoV-2 infection also resulted in the upregulation of genes involved in viral responses, with pathway analysis predicting BRD4 among the significant upstream regulators of these differentially expressed genes. Similar findings were made with CS-treated hCOs, supporting the translational validity of their *ex vivo* organoid model and the choice of components to emulate CS in the system. Interestingly, the author failed to detect the presence of SARS-CoV-2 in the hearts of the mice, suggesting that indirect effects, such as CS, resulted in transcriptome remodeling in the heart. In parallel studies, INCB054329 reduced cytokine expression and prevented mortality and systolic dysfunction in mice acutely treated with LPS to mimic CS.

Finally, moving back to the hCO system, the authors show that multiple BET inhibitors can block CS-induced relaxation impairment, including apabetalone, elevating the translational significance of the work. Indeed, apabetalone is the only BET inhibitor to be tested in a Phase 3 trial, being assessed for its ability to reduce major cardiovascular events in > 2400 individuals with combined acute coronary syndrome (ACS), type 2 diabetes (T2D), and low LDL levels. While apabetalone failed to diminish ischemic cardiovascular events in this patient population, the BET inhibitor was found to be well-tolerated, and secondary subgroup analyses revealed a reduction in hospitalizations for heart failure in patients with T2D and recent ACS^[8]^, and fewer heart failure-related hospitalizations in patients with chronic kidney disease and T2D^[9]^. In addition, apabetalone and a related compound also reduced ACE2 expression and SARS-CoV-2 infection in the hCO model, highlighting another possible mechanism by which this approach could improve cardiac outcomes in COVID-19 patients.

As with any powerful study, the work of Mills *et al.*^[5]^ raises new questions and avenues for future investigation. As pointed out by the authors, the inhibitors used in their study target all BET family members so, while BRD4 appears to be the culprit that drives CS-induced DD since its knockdown in the hCO model improved relaxation, the involvement of other BET family members cannot be ruled out. Parallel knockdown of BRD2, BRD3, and BRD4, alone or in combination, in the hCO model would address the importance of specific BET family members to the pathogenesis of CS-induced DD and could guide future efforts to target specific members of the family as a safer approach than pan-BET inhibition.

A strength of the work by Mills *et al.*^[5]^ was the use of an advanced version of their in-house, self-organizing cardiac organoid system, which was created using human pluripotent stem cell (hPSC)-derived cells mixed in cell culture plates. Comparative single-cell RNA-sequencing of hCOs revealed significant overlap in cell-type clustering relative to the healthy human heart. Furthermore, CS treatment of hCOs induced a consistent pathological transcriptome signature and increased relaxation time, emulating DD. Nonetheless, since this model was derived from *in vitro* differentiated cells, lacks comorbidities such as aging and diabetes, and is not influenced by distal organs, it is unlikely reflective of the extreme heterogeneity in phenotype and response to drugs seen in HFpEF patients. As large datasets emerge from analyses of cardiac samples from patients with DD and/or COVID-19, cell-type composition shifts and BRD4-regulated gene signatures seen in CS-treated hCOs can be cross-validated with patient samples to address further the translational value of the organoid model.

The mechanism(s) by which BET inhibition ameliorates CS-induced DD remains a mystery, and we are left to assume that, in COVID-19 patients, BRD4 is activated upon phosphorylation to stimulate pathological gene expression that results in cardiac relaxation impairment [Figure 1]. Evidence is emerging for widespread transcriptional remodeling in the SARS-CoV-2 infected human heart, and BET proteins almost certainly contribute to this process. Nonetheless, how the BET inhibitor-mediated changes in gene expression observed in the current study culminate in improved cardiac relaxation is unclear, as the expression of markers of fibrosis, which is one driver of DD, were decreased hCOs treated with CS. Future studies should address whether BET inhibitor treatment improves myofibril relaxation, titin compliance and/or calcium handling in CS-treated hCOs to address further the mechanism(s) of the efficacy of the compounds in this model. Additionally, since DD was not observed in SARS-CoV-2-infected or LPS-treated mice, additional work is needed in true models of DD to determine whether BET inhibition is a viable approach for treating this cardiac abnormality. It will also be interesting to examine whether BRD4 phosphorylation is altered in the hearts of humans suffering from DD and if so, to determine the responsible kinases and functional consequences.

Despite these open questions, Mills *et al.*^[5]^ are commended for quickly attacking an enormous global health problem using a battery of state-of-the-art methods. Their findings suggest an intimate relationship between COVID-19 and BRD4 and demonstrate that BET bromodomain inhibition can reduce cardiac stress and improve relaxation of the heart in the face of a cytokine storm triggered by SARS-CoV-2 infection.

## Figures and Tables

**Figure 1.**
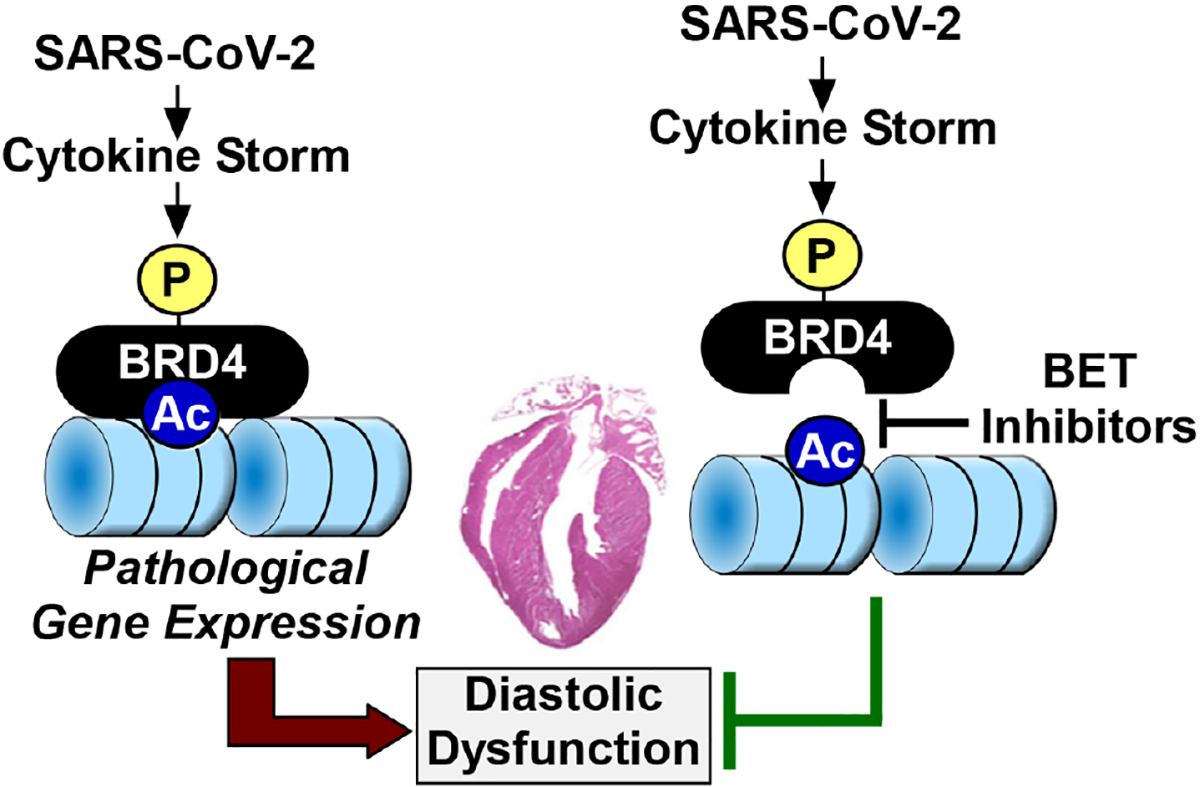
A model for BRD4-induced diastolic dysfunction in COVID-19 patients. A cytokine storm elicited by SARS-CoV-2 infection triggers phosphorylation and activation of BRD4, resulting in changes in gene expression that culminate in diastolic dysfunction. BET inhibitors prevent the binding of BRD4 to acetyl-histones in chromatin, thereby preventing cytokine storm-driven diastolic dysfunction. BRD4: Bromodomain-containing protein 4; BET: bromodomain and extra-terminal.
